# Endodontic Radiopacifying Application of Barium Titanate Prepared through a Combination of Mechanical Milling and Heat Treatment

**DOI:** 10.3390/ma16237270

**Published:** 2023-11-22

**Authors:** Hsiu-Na Lin, Wei-Wen Chen, Chun-Chun Hsu, May-Show Chen, Pei-Jung Chang, Wei-Min Chang, Fang-Hao Zhang, Chin-Yi Chen, Pee-Yew Lee, Chung-Kwei Lin

**Affiliations:** 1 Research Center of Digital Oral Science and Technology, College of Oral Medicine, Taipei Medical IUniversity, Taipei 110, Taiwan; tiffanylin1214@gmail.com (H.-N.L.); maychen@tmu.edu.tw (M.-S.C.); peronchang@tmu.edu.tw (P.-J.C.); weiminchang@tmu.edu.tw (W.-M.C.); chencyi@fcu.edu.tw (C.-Y.C.); 2 Department of Dentistry, Chang Gung Memorial Hospital, Taipei 105, Taiwan; 3 School of Dentistry, College of Oral Medicine, Taipei Medical University, Taipei 110, Taiwan; 4 Center of Dental Technology, Chang Gung Memorial Hospital, Linkou, Taoyuan 333, Taiwan; curecure2@cgmh.org.tw; 5 School of Respiratory Therapy, College of Medicine, Taipei Medical University, Taipei 110, Taiwan; chunhsu@tmu.edu.tw; 6 Graduate Institute of Medical Sciences, College of Medicine, Taipei Medical University, Taipei 110, Taiwan; 7 Division of Pulmonary Medicine, Department of Internal Medicine, Taipei Medical University Hospital, Taipei 110, Taiwan; 8 Division of Prosthodontics, Department of Dentistry, Taipei Medical University Hospital, Taipei 110, Taiwan; 9 Graduate Institute of Manufacturing Technology, National Taipei University of Technology, Taipei 106, Taiwan; 10 School of Oral Hygiene, College of Oral Medicine, Taipei Medical University, Taipei 110, Taiwan; 11 Department of Optoelectronics and Materials Technology, National Taiwan Ocean University, Keelung 202, Taiwan; jeff26225@gmail.com; 12 Department of Materials Science and Engineering, Feng Chia University, Taichung 407, Taiwan; 13 School of Dental Technology, College of Oral Medicine, Taipei Medical University, Taipei 110, Taiwan

**Keywords:** mineral trioxide aggregates, radiopacifier, barium titanate, mechanical milling, radiopacity, discoloration, setting time

## Abstract

Mineral trioxide aggregates (MTA) are commonly used as endodontic filling materials but suffer from a long setting time and tooth discoloration. In the present study, the feasibility of using barium titanate (BTO) for discoloration and a calcium chloride (CaCl_2_) solution to shorten the setting time was investigated. BTO powder was prepared using high-energy ball milling for 3 h, followed by sintering at 700–1300 °C for 2 h. X-ray diffraction was used to examine the crystallinity and crystalline size of the as-milled and heat-treated powders. MTA-like cements were then prepared using 20–40 wt.% BTO as a radiopacifier and solidified using a 0–30% CaCl_2_ solution. The corresponding radiopacity, diametral tensile strength (DTS), initial and final setting times, and discoloration performance were examined. The experimental results showed that for the BTO powder prepared using a combination of mechanical milling and heat treatment, the crystallinity and crystalline size increased with the increasing sintering temperature. The BTO sintered at 1300 °C (i.e., BTO-13) exhibited the best radiopacity and DTS. The MTA-like cement supplemented with 30% BTO-13 and solidified with a 10% CaCl_2_ solution exhibited a radiopacity of 3.68 ± 0.24 mmAl and a DTS of 2.54 ± 0.28 MPa, respectively. In the accelerated discoloration examination using UV irradiation, the color difference was less than 1.6 and significantly lower than the clinically perceptible level (3.7). This novel MTA exhibiting a superior color stability, shortened setting time, and excellent biocompatibility has potential for use in endodontic applications.

## 1. Introduction

Canal treatment is an important clinical practice in endodontics where mineral trioxide aggregate (MTA) serves as a dental filling and radiopacifying material for lateral perforations, apexification, direct pulp capping, and root end filling [1,2,3,4,5,6]. The long setting time and tooth discoloration are key issues to be addressed for MTAs [7,8,9]. The setting time is immediate, and discoloration occurs after treatment.

Typically, MTA consists of 80% Portland cement and 20% radiopacifier (Bi_2_O_3_) mixed with a solution for solidification. Setting times are directly related to the Portland cement and solidifying solution [2,10]. The modification of Portland cement and use of alternative calcium-silicate-based cements have been attempted [11,12,13,14,15]. Various solidifying solutions that include calcium chloride, calcium nitrate, calcium formate [16,17], calcium lactate gluconate [18], disodium hydrogen phosphate [19], tannic acid [20], silk fibroin [21], etc., were investigated to shorten the setting times. For the most part, tooth discoloration is caused by blood contamination [8,22] and the radiopacifier Bi_2_O_3_ [9,23]. Alternative radiopacifiers, such as ZrO_2_ and Ta_2_O_5_, have been used in commercial products to minimize tooth discoloration [24,25].

Recently, bioceramics for endodontic applications have been reviewed [26,27]. Among them, barium titanate (BaTiO_3_, BTO), which exhibits a perovskite crystalline structure, has been widely used in dielectric and ferroelectric applications [28,29,30]. The phase transition and dielectric performance of BTO and erbium-doped BTO were elucidated by Leyet et al. [31]. The positive temperature coefficient’s resistivity effect on the ferroelectric-paraelectric phase transition was addressed. The application of BTO nanoparticles in various biomedical fields has been attempted [32,33,34]. For instance, Choi et al. reported the effects of barium titanate addition on the radiopacity and biocompatibility of tricalcium silicate-based bioceramics for bone regeneration [33]. This indicates that BTO is a potential candidate endodontic radiopacifying and filling material.

Barium titanate can be prepared using various wet chemical techniques [30], including the solvothermal method [35], hydrothermal synthesis [36,37], sol–gel process [38,39], and a physical ball milling process [40,41]. The high-energy ball milling process is a facile method used to synthesize various materials, such as metastable amorphous materials, extended solid solutions, intermetallic compounds, nanocrystalline powders, etc. [42,43,44]. BTO powder can be synthesized by milling BaCO_3_ and TiO_2_, followed by a high-temperature treatment [40,45,46]. When using BaO and TiO_2_ as starting materials, high-energy ball milling may induce a mechanochemical reaction and result in BaTiO_3_ formation [41].

Novel MTA with the combination of a suitable radiopacifier and solidification solution is an attractive research and development topic [10]. Alternative radiopacifiers including oxides (ZrO_2_ and Ta_2_O_5_) and perovskite structure materials (BaZrO_3_ and CaZrO_3_) have been used in commercial endodontic products [47]. Though the applications of BTO in medical fields have been addressed, the feasibility of using BTO in dentistry is low. In the present study, barium titanate was prepared by combining high-energy ball milling with high-temperature sintering at 700–1300 °C. The as-prepared BTO was solidified using various concentrations of calcium chloride solution (0–30%) to prepare MTA-like cements. The effects of sintering temperature, the amount of BTO addition, and the concentration of solidifying solution on the performance of the MTA-like cements were investigated to determine their potential endodontic application.

## 2. Materials and Methods

### 2.1. Preparation and Characterization of Barium Titanate

Commercially available BaCO_3_ (<5 µm, purity 99.9%, Ultimate Materials Technology Co., Ltd., Hsinchu, Taiwan) and TiO_2_ powders (<45 µm, purity 99.99%, Ultimate Materials Technology Co., Ltd., Hsinchu, Taiwan) were used as the starting materials for mechanical milling. A SPEX 8000D shaker ball mill (Fisher Scientific, Ottawa, ON, Canada) positioned in an Ar-filled glove box was used for this process [48]. Within the environment-controlled glove box, the total oxygen and water concentration was kept lower than 100 ppm. The starting powder (BaCO_3_ and TiO_2_ in an equal molar concentration with a total weight of 6 g) and 7 mm Cr-steel balls (~30 g) were canned in a SKH 9 high-speed steel vial (40 mm and 50 mm in diameter and height, respectively) for 3 h of mechanical milling treatment. The as-milled powder was then sintered, respectively, at 700, 900, 1100, and 1300 °C for 2 h. The heat-treated powder was coded, respectively, as BTO-7, -9, -11, and -13 and examined using an X-ray diffractometer (Bruker AXS GmbH-D2 PHASER, Billerica, MA, USA) with Ni-filtered Cu Kα emission. The crystalline size of the as-prepared BTO powder was calculated according to Scherrer’s formula with a shape factor (k) equal to 0.9 using the Rietveld fitting method with the XRD analysis software EVA (Bruker-AXS DiffracEVA, version 6.0, Bruker, WI, USA) [49,50].

### 2.2. Preparation and Characterization of MTA-Like Cements

MTA-like cements were prepared by mixing 80 wt.% Portland cement with 20 wt.% BTO powder using a benchtop ball mill (Retsch PM100, Haan, Germany) for 10 min. For solidification, the mixed powder was mixed with deionized water or a 10, 20, and 30% calcium chloride solution using a powder-to-water ratio equal to 3. The pastes were then placed into a mold (10 mm diameter and 1 mm thickness for radiopacity; 6 mm diameter and 5 mm height for diametral tensile strength; *n* = 6 for both experiments). After solidification, the MTA-like cements were placed in an environment-controlled incubator (37 °C with 100% relative humidity) for another 24 h to simulate the oral environment. The detailed experimental procedures are available elsewhere [51].

Radiopacity was examined using a dental X-ray system (VX-65; Vatech Co, Yongin Si Gyeonggi-Do, Republic of Korea) in which a radiographic film (Koadak CR imaging plate size 2; Eastman-Kodak Co, Rochester, NY, USA) was located at a distance of 30 cm. The X-ray equipment was operated at a voltage of 62 kV, a current density of 10 mA, and an exposure time of 0.64 s. X-ray images of six samples and a reference aluminum step-wedge were taken simultaneously and analyzed using Image J software (version 1.53s, Wayne Rasband, National Institutes of Health, Bethesda, MD, USA). The diametral tensile strength (DTS) was measured with a universal test machine (CY-6040A8, Chun Yen testing machines, Taichung, Taiwan) using a crosshead speed of 6.0 mm/min and calculated using DTS = 2F/πbw, where F is the maximum applied load (N) and b and w are the diameter (mm) and the height (mm) of the sample, respectively.

### 2.3. Setting and Discoloration of MTA-like Cements

MTA-like cements (*n* = 3) set by adding deionized water with 10, 20, and 30% CaCl_2_ solution were placed into an acrylic mold with a diameter and height of 6 mm and 5 mm, respectively. Both the DI water and CaCl_2_ solutions were colorless and transparent. The initial and final setting times were determined with a Vicat needle (Jin-Ching-Her Co. Ltd., Yunlin County, Taiwan) that was equipped with a movable rod weighing 300 g and a diameter measuring 1 mm. The depth of impression was measured and the initial and final setting times were determined based on the depth, measuring less than 1 mm and zero, respectively.

Discoloration was applied to the disc samples (10 mm diameter and 1 mm thickness) by immersing them in 2 mL glycerin (Wako, Osaka, Japan) for 15 min and exposing the soaked samples to UV irradiation [52]. A UV curing machine (Phrozen Cure V2, Hsinchu, Taiwan) with UV-LEDs (365 nm, 385 nm, and 405 nm, 60 W in total) was used for the discoloration experiments [51]. Excepting the practical photographs for observation, a digital dental colorimeter (OptiShade Styleitaliano, St-Imier, Switzerland) was used to obtain the L*a*b* values of the exposed samples (*n* = 6). The color differences between the exposed and unexposed samples were calculated using △E_00_ according to the CIE standard [53].

### 2.4. Biocompatibility Assay of BTO-13-Supplemented MTA-like Cement

The samples for testing biocompatibility were prepared as discs (10 mm diameter and 1 mm thickness) similar to those used for the radiopacity and discoloration tests. The biocompatibility of the BTO-13-supplemented MTA-like cements solidified with various solutions was measured using a CCK8 mitochondria activity assay (Donjindo, Kumamoto, Japan), followed by the ISO-10993-5 standard [54] protocol. The L929 cells were cultured in a minimal essential medium (MEM, Gibco, Thermo Fisher Scientific Inc., Waltham, MA, USA) supplemented with 10% of fetal bovine serum (FBS, Sigma-Aldrich, Merck, Burlington, MA, USA) and 1% penicillin/streptomycin (PS, Gibco) and cultured in 5% CO_2_ at 37 °C. 

In this experiment, L929 cells without extracts from BTO-13-supplemented MTA-like cement served as the control group, and each sample was tested with four replicates. The L929 cells were seeded at a density of 10^4^ per well in a 96-well plate. Briefly, the L929 cells were cultured in extracts from the BTO-13-supplemented MTA-like cement soaked for 24 h. The fresh culture medium with the 10% CCK8 solution were replaced for an additional 2 h of incubation and the absorbance was measured at 450 nm (Multiskan FC, Thermo Fisher Scientific Inc., Waltham, MA, USA). The cell morphologies were observed using a ZEISS AXIOVERT 200 inverted phase contrast microscope (ZEISS, Oberkochen, Germany).

## 3. Results and Discussion

### 3.1. Synthesis of Barium Titanate Powder

**Figure 1:**
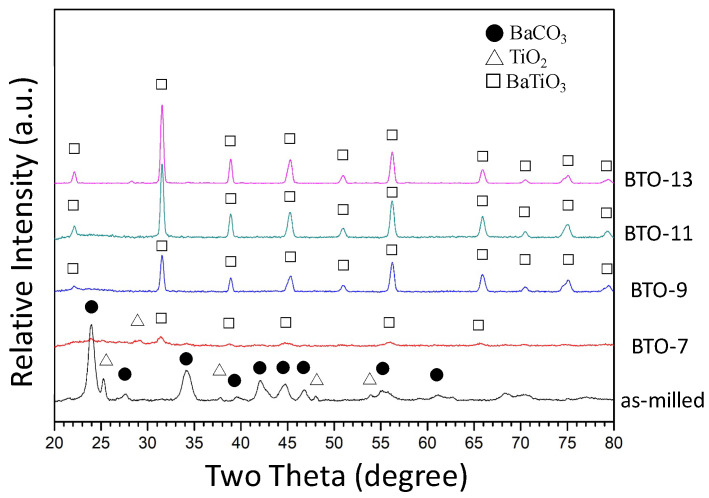
X-ray diffraction patterns of as-milled and heat-treated powder.

Figure 1 shows the X-ray diffraction patterns of the as-milled powder after 3 h of milling and the heat-treated powder sintered at 700, 900, 1100, and 1300 °C for 2 h, respectively. As shown by the bottom black curve in Figure 1, the XRD pattern revealed that the powder milled for 3 h exhibited a mixture of BaCO_3_ (orthorhombic phase, ICDD PDF card No. 05-0378) and TiO_2_ phases (tetragonal phase, ICDD PDF card No. 04-0477). Only the refined starting powder (i.e., BaCO_3_ and TiO_2_) without the formation of the desired BaTiO_3_ phase was observed. Since both BaCO_3_ and TiO_2_ were brittle, it was suggested that the starting powders were cracked into small pieces, entangled with each other, and continuously refined with the increasing milling time [55]. The relatively high energy input during ball milling did not trigger a mechanochemical reaction between BaCO_3_ and TiO_2_ for the formation of BaTiO_3_. The reaction is shown below:

BaCO_3_ + TiO_2_ → BaTiO_3_ + CO_2_

The formation of BaTiO_3_ is accompanied by the byproduct CO_2_ gas. The energy input during the mechanical milling process increases the temperature of the environment. According to the ideal gas law, the pressure will increase and hinder the formation of CO_2_ and BaTiO_3_. Therefore, the desired BaTiO_3_ phase cannot be synthesized through high-energy ball milling. The refined starting powder, however, was preferable for the following heat treatment. The reaction between BaCO_3_ and TiO_2_ was observed after sintering at 700 °C, as shown by the red curve (BTO-7) in Figure 1. Pavlović et al. [45] synthesized BaTiO_3_ using a planetary ball mill. BaTiO_3_ was prepared by milling a BaCO_3_ and TiO_2_ powder mixture for 1.5 h, followed by heat treatment at 1200 °C. Othman et al. [46] increased the milling time to 7.5 h, and the sintering temperature was lowered to 900 °C. In the present work, using the high-energy SPEX 8000D ball mill, BaTiO_3_ powder was synthesized by sintering the powder mixture milled for 3 h at 700 °C. This suggests that a high-energy ball milling treatment can refine the starting powder and lower the sintering temperature for the formation of BaTiO_3_. The higher the sintering temperature, the sharper the diffraction peaks (as shown by BTO-9, -11, and -13), and the better the crystallinity of BaTiO_3_ (cubic phase, ICDD PDF card No. 31-0174).

**Figure 2:**
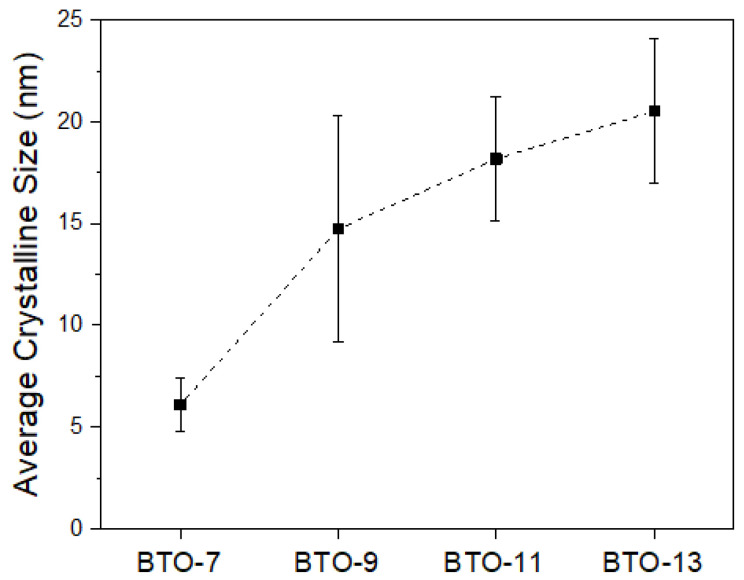
Average crystalline size of heat-treated barium titanate powder.

The as-prepared BaTiO_3_ powder is destined to be used as an endodontic radiopacifying material, and thus, the crystalline size may be an important issue for solidification. Figure 2 shows the average crystalline size of the as-milled powder after sintering at 700, 900, 1100, and 1300 °C for 2 h, respectively. It can be observed that not only the crystallinity (revealed by the XRD pattern in Figure 1) but also the average crystalline size increased with the increasing sintering temperature. The average crystalline size was 6.1 ± 1.3 nm for BTO-7 and gradually increased to 20.5 ± 3.6 nm for BTO-13.

As discussed in Figure 1 and Figure 2, high-energy ball milling can effectively refine the crystalline size of the starting powders and lower the sintering temperature to 700 °C. At 700 °C, the reaction between the BaCO_3_ and TiO_2_ powder mixture, however, was not finished within 2 h. The powder mixture exhibited a major BTO phase (~74%) and minor BaCO_3_ and TiO_2_ phases. The crystalline size of the resulting BTO was 6.1 nm. Complete BTO formation, however, was noticed after sintering at 900 °C for 2 h. For sintering, the crystalline size increases with either increasing temperature or time. It is evident that within the same sintering time of 2 h, the crystalline size increased from 6.1 nm for BTO-7 to 14.7, 18.2, and 20.5 nm for BTO-9, -11, and -13, respectively.

### 3.2. BaTiO_3_ as Radiopacifier for MTA

**Figure 3:**
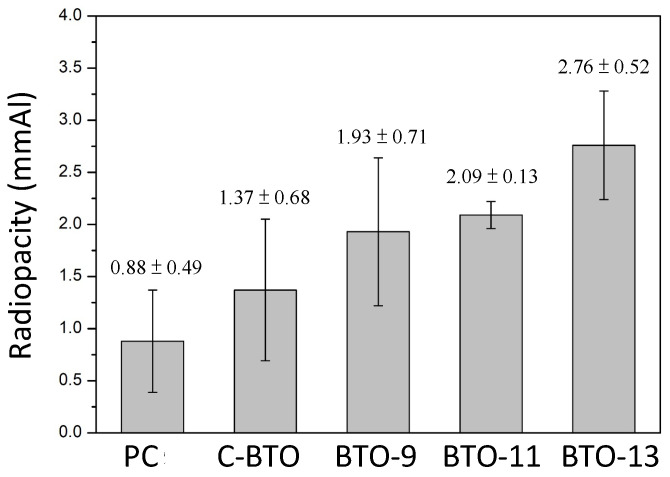
Radiopacity of various MTA-like cements prepared using Portland cement (PC), commercial barium titanate (C-BTO), and BTO-9, BTO-11, and BTO-13 powders.

The obtained BaTiO_3_ powder was used as the radiopacifier for mineral trioxide aggregates (MTAs). MTA-like cements were prepared, and the corresponding radiopacities were measured, as shown in Figure 3. Though not shown here, the MTA-like cement prepared using BTO-7 was not very successful, probably due to its fine crystalline size and need for more solution for solidification. Without a radiopacifier, the MTA-like cement prepared using Portland cement exhibited a low radiopacity of 0.88 ± 0.49 mmAl. It increased to 1.37 ± 0.68 mmAl with the addition of the commercially available BaTiO_3_ (coded as C-BTO). Using the BaTiO_3_ prepared in the present study, the radiopacity improved to 1.93 ± 0.71, 2.09 ± 0.13, and 2.76 ± 0.52 mmAl for BTO-9, BTO-11, and BTO-13, respectively. Since the MTA-like cements were prepared by mixing 80% Portland cement and 20% radiopacifier, the radiopacity performance was mainly affected by the radiopacifier (crystalline phases and size) and solidifying solution. As shown in Figure 2, the crystalline size for the sol–gel-treated BTO was in the nano-sized range (6.1–20.5 nm). BTO-7 (6.1 nm) was too small to have enough wetting. The others (14.7–20.5 nm) were more suitable for the solidifying solution to wet the powder. A large crystalline size was beneficial for the radiopacity performance. The radiopacity increased with the increasing sintering temperature. However, none of the values satisfied the ISO 6876:2012 [56] requirement (3 mmAl). This can be attributed to the relatively low atomic numbers of Ba and Ti. 

**Figure 4:**
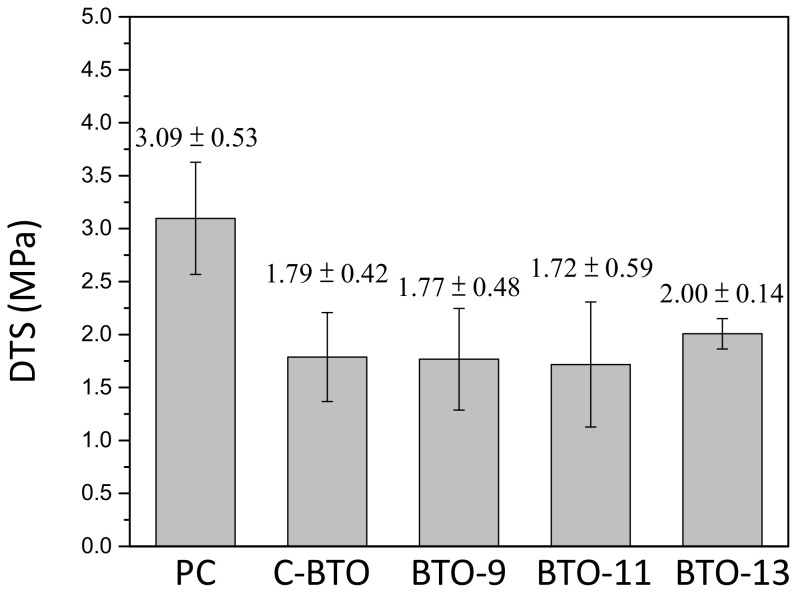
Diametral tensile strength of various MTA-like cements prepared using Portland cement (PC), commercial barium titanate (C-BTO), and BTO-9, BTO-11, and BTO-13 powders.

The mechanical properties of the MTA-like cements were evaluated using a diametral tensile test, and Figure 4 shows the corresponding results for the MTA-like cements presented in Figure 3. It can be noted that the MTA-like cements prepared using Portland cement possessed the highest diametral tensile strength of 3.09 ± 0.53 MPa, which was significantly higher than the values for the other samples. The one using C-BTO exhibited the lowest diametral tensile strength (1.79 ± 0.42 MPa), whereas the DTS values were 1.77 ± 0.48 MPa, 1.72 ± 0.59 MPa, and 2.00 ± 0.14 MPa for BTO-9, BTO-11, and BTO-13, respectively. Though no significant difference can be noticed for the BTO samples, BTO-13 exhibited not only a slightly higher DTS value but also a smaller deviation.

**Figure 5:**
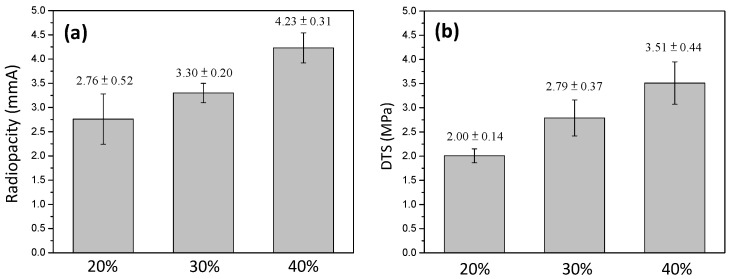
(**a**) Radiopacity and (**b**) diametral tensile strength of MTA-like cements prepared using 20, 30, and 40% BTO-13 powder.

As shown above, the MTA-like cements prepared using BTO-13 powder exhibited the best radiopacity (2.76 ± 0.52 mmAl) and DTS (2.00 ± 0.14 MPa) performance. The radiopacity, however, did not meet the required 3 mmAl. This is similar to the MTA-like cements with zirconia as the radiopacifier [51]. In order to reveal the therapeutic outcome of endodontic treatment, the radiopacity is highly important and must be larger than 3 mmAl to determine the differences between the MTAs and tooth (which has a relatively low radiopacity). Since the radiopacity increases with the increasing amount of radiopacifier, Figure 5 shows the radiopacity and corresponding DTS of the MTA-like cements prepared using 20–40% BTO-13 powder. It can be noted that the radiopacity (Figure 5a) increased with the increasing amount of BTO-13 and was 2.76 ± 0.52, 3.30 ± 0.20, and 4.23 ± 0.31 mmAl for 20, 30, and 40% BTO-13, respectively. A similar trend can be observed for the DTS results. As shown in Figure 5b, the diametral tensile strength of 20, 30, and 40% BTO-13 was 2.00 ± 0.14, 2.79 ± 0.37, and 3.51 ± 0.44 MPa, respectively. The radiopacity and DTS results suggested that MTA-like cement with 30% or 40% BTO-13 as a radiopacifier can be used as an alternative MTA. However, it should be pointed out that it was difficult to manipulate the paste with 40% BTO-13 during the preparation of MTA-like cements. Thus, MTA-like cements with 30% BTO-13 were examined further for their setting time, discoloration, and biocompatibility.

### 3.3. Effect of Calcium Chloride Solution on Setting and Discoloration

**Figure 6:**
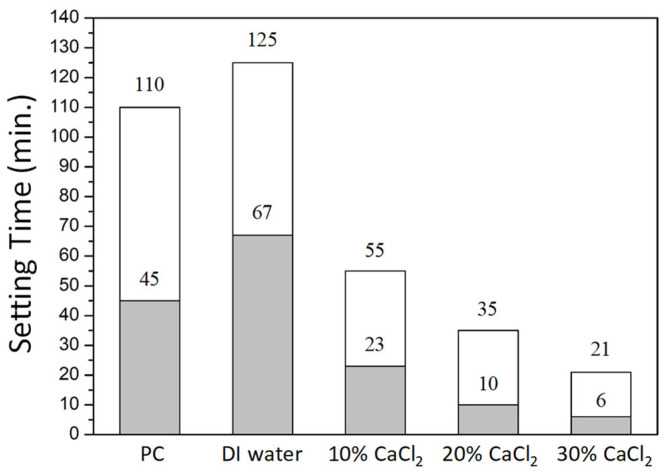
Initial (grey color) and final (white color) setting time for MTA-like cements prepared using BTO-13 powder and solidified with deionized water, and 10–30% CaCl_2_ solution. Portland cement (PC) solidified using deionized water is also given for comparison.

In addition to the radiopacity and diametral tensile strength, setting time and discoloration are also important factors in clinical application. Figure 6 shows the setting time for the MTA-like cement prepared using 30% BTO-13 and solidified with deionized water and 10–30% CaCl_2_ solution, respectively. It can be observed that the MTA-like cement prepared using only Portland cement exhibited relatively long setting times, where the initial and final setting times were 45 and 110 min, respectively. After adding the 30% BTO-13 radiopacifier, the initial and final setting times extended, respectively, to 67 and 125 min, which may not be suitable for practical application. Using a calcium chloride solution to solidify MTA-like cements can significantly shorten the setting time. The initial setting time was shortened, respectively, to 23, 10, and 6 min, whereas the final setting time was 55, 35, and 21 min for the 10%, 20%, and 30% CaCl_2_ solutions.

**Figure 7:**
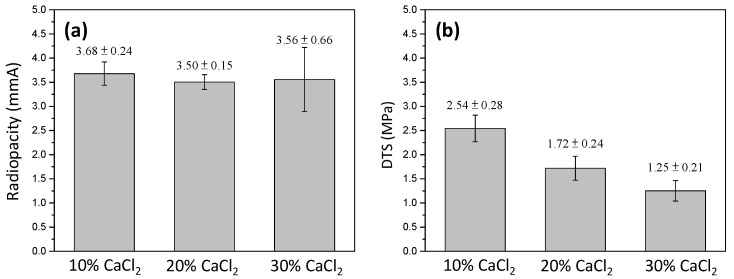
(**a**) Radiopacity and (**b**) diametral tensile strength of MTA-like cements prepared by using 30% BTO-13 and solidified with 10–30% CaCl_2_ solution, respectively.

Using the CaCl_2_ solution, the corresponding radiopacity and DTS were examined and are presented in Figure 7. It can be noted that the radiopacity did not exhibit a significant difference. Compared to that solidified with deionized water (3.30 ± 0.20 mmAl, Figure 5), the radiopacity slightly increased to 3.68 ± 0.24, 3.50 ± 0.15, and 3.56 ± 0.66 mmAl for the 10, 20, and 30% CaCl_2_ solutions, respectively. In contrast, the DTS continuously decreased with the increasing concentration of CaCl_2_. It decreased from 2.79 ± 0.37 MPa (DI water, 0% CaCl_2_) to 2.54 ± 0.28, 1.72± 0.24, and 1.25 ± 0.21 MPa for the 10, 20, and 30% CaCl_2_ solutions. The higher the CaCl_2_ concentration, the lower the DTS. This suggests that 10% CaCl_2_ is the optimal solidifying solution.

**Figure 8:**
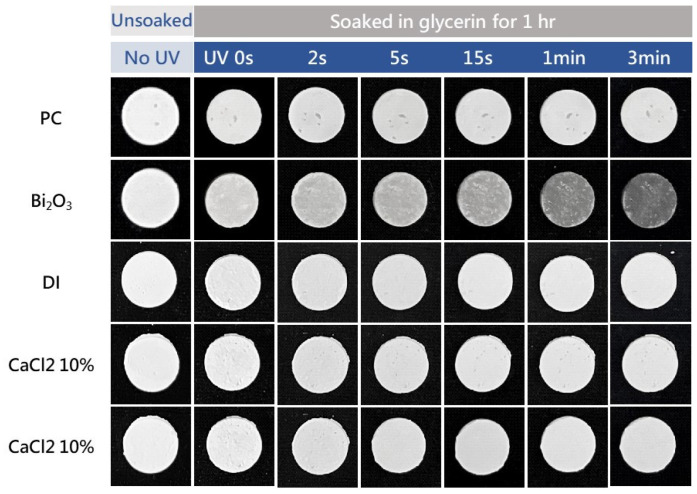
Photos of MTA-like cements prepared using BTO-13 powder and solidified with deionized water, as well as 10% and 20% CaCl_2_ solutions. Portland cement (PC) and PC with Bi_2_O_3_ solidified using deionized water are also given for comparison.

**Figure 9:**
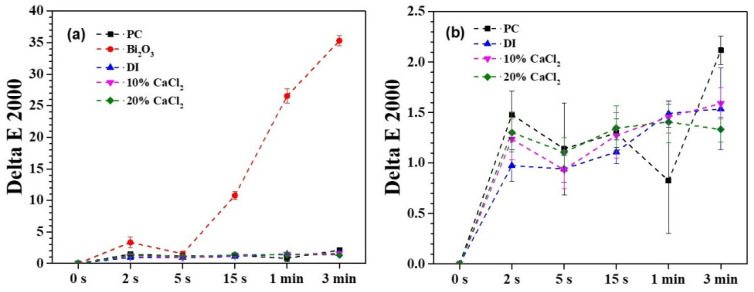
(**a**) Full scale and (**b**) partial scale for ΔE_00_ values of MTA-like cements prepared using BTO-13 powder and solidified with deionized water, as well as 10% and 20% CaCl_2_ solutions. Portland cement (PC) and PC with Bi_2_O_3_ solidified using deionized water are also given for comparison.

An accelerated discoloration examination was performed using UV irradiation experiments to simulate the aesthetics after endodontic therapy for a period of time (approximately one month) [51,52]. Figure 8 shows the photos of a series of MTA-like cements before and after UV irradiation. The photos in the leftmost column show the MTA-like cements before the experiment. A relatively light gray color of all the samples can be observed, and no significant difference can be distinguished. After soaking in glycerin for 1 h (the second from the left), a slight color variation can be observed. Only the sample prepared using the Bi_2_O_3_ radiopacifier (the second from the top) exhibited perceptible discoloration when treated with UV irradiation. Limited discoloration for the rest of the samples was observed. This color variation can be examined further using the CIE L*a*b* values of these samples, and Figure 9 shows the corresponding results. It can be noted that the MTA-like cement with the Bi_2_O_3_ radiopacifier exhibited a significant difference when compared with the other samples. As shown in Figure 9a, the ΔE_00_ was 10.8 after a very short UV irradiation period of 15 s. This significantly exceeded the clinically perceptible level of 3.7 [57]. The ΔE_00_ increased continuously to 26.5 after 1 min. The UV irradiation reached 35.3 at the end of the experiment (3 min). In order to observe the variation in the other samples, Figure 9b shows the other samples without the Bi_2_O_3_-supplemented MTA-like cement. In general, all the samples started showing limited discoloration after UV irradiation. The ΔE_00_ fluctuated with the increasing irradiation time. The MTA-like cement without a radiopacifier (i.e., PC) fluctuated between 1.5 and 2.1. With BTO-13, solidified using deionized water and 10% and 20% CaCl_2_ solutions, the ΔE_00_ ranged from 1.0 to 1.6, without any significant difference, and these values were much lower than the clinically perceptible level [51,57]. The MTA-like cements without Bi_2_O_3_ exhibited color stability. Table 1 summarizes all the ΔE_00_ results for the discoloration experiments.

### 3.4. Biocompatibility of MTA-like Cements

**Figure 10:**
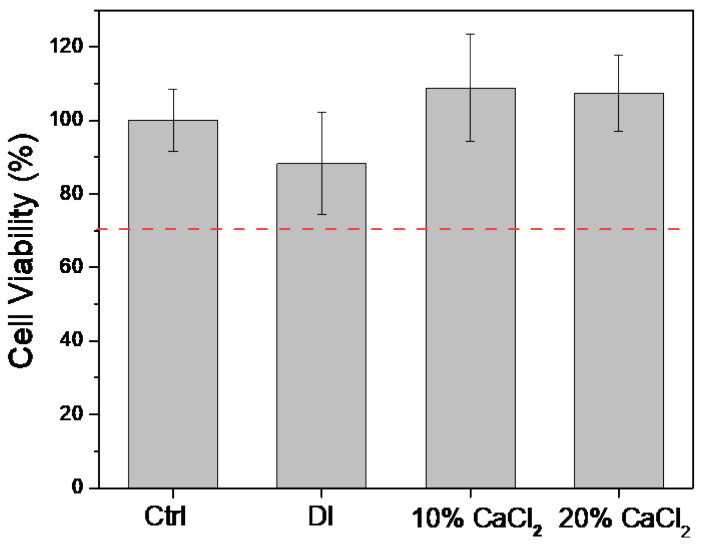
Cell viability of L929 cells immersed in the extracts of 30% BTO-13-supplemented MTA-like cements solidified with deionized water (DI) and 10% CaCl_2_ and 20% CaCl_2_ solutions.

Before the practical application of this novel endodontic radiopacifying material, the biocompatibilities of the MTA-like cements were evaluated according to the ISO 10993-5 standard [54]. The L929 cells were treated with the extracts from MTA-like cements prepared by adding deionized water with 10% and 20% CaCl_2_ solutions. The biocompatibility was examined using the CCK8 kit, and the results for cell viability are shown in Figure 10. It can be noted that all the examined samples were biocompatible, with a cell viability higher than 70% (ISO 10993-5 standard [54]). This results also indicated that, compared to the cell viability of the control group (100 ± 8%), the MTA-like cement solidified with deionized water exhibited a cell viability of 88% ± 14% that increased to 109 ± 15% and 107 ± 10% when using 10% and 20% CaCl_2_, respectively. This shows a similar trend to that reported by Pinto et al. [58], who noted that calcium ions can effectively promote cell proliferation. This improved cell biocompatibility and proliferation may be beneficial for these novel MTAs’ use in endodontic lateral perforation and other bone regeneration applications. 

**Figure 11:**
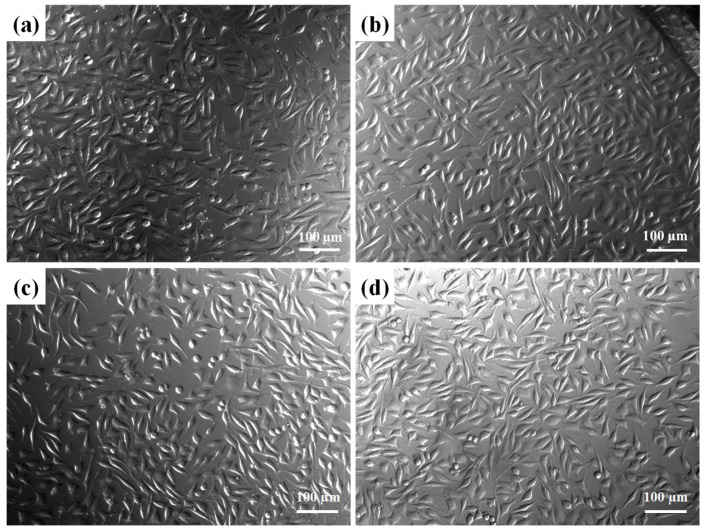
L929 cell morphology of BTO13-30 with different extracts, including (**a**) Ctrl, (**b**) PC, (**c**) 10% CaCl_2_, (**d**) and 20% CaCl_2_ immersion for 24 h.

Furthermore, on the basis of the biocompatibility test, the morphology of the L929 cells corresponding to the samples in Figure 9 was examined under a microscope. As shown in Figure 11, the extracts of the BTO-13-supplemented MTA-like cements solidified with deionized water and the 10% and 20% CaCl_2_ solutions did not change the L929 cell shape or cause any damage. Their cell appearances (Figure 11b–d) are similar to those of the control (Figure 11a), exhibiting spindle-like, epithelial-like, stellate, and round shapes.

It should be pointed out that BTO can be prepared using various techniques, including physical solid-state synthesis (as in the present work) and chemical wet processes [30,35,36,37,38,39]. Slight differences in the synthesized BTO can be expected. In the present work, we demonstrated the properties and the feasibility of this novel MTA. Further investigations including continuous modifications of the present formula, to further improve its performance, and in vivo animal experiments before clinical practice are in progress.

As demonstrated above, the MTA-like cement prepared by adding 30% BTO-13 and solidified with 10% CaCl_2_ was optimal for endodontic application, with a suitable radiopacity (3.68 ± 0.24 mmAl), DTS (2.54 ± 0.28 MPa), appropriate initial and final setting times (55 and 23 min, respectively), no discoloration, and superior biocompatibility. It is a new potential MTA for use in endodontic treatment.

## 4. Conclusions

In the present study, barium titanate powder was prepared using a combination of mechanical milling for 3 h and heat treatment at 700–1300 °C for 2 h. The higher the sintering temperature was, the larger the crystalline size and the better the crystallinity were. The radiopacity of the MTA-like cements increased with the increasing sintering temperature, whereas no significant difference could be observed in the DTS. With 30% BTO-13 addition, the MTA-like cements exhibited a radiopacity of 3.30 ± 0.20 mmAl and a DTS of 2.79 ± 0.37 MPa. Using CaCl_2_ as a solidifying solution, the setting time could be shortened without decreasing the radiopacity, whereas the DTS decreased with the increasing concentration of CaCl_2_. This suggests that MTA-like cement prepared by adding 30% BTO-13 and solidified using a 10% CaCl_2_ solution is optimal. It exhibited a radiopacity of 3.68 ± 0.24 mmAl, a DTS of 2.54 ± 0.28 MPa, and initial and final setting times of 55 and 23 min, respectively. This novel MTA also possessed excellent color stability and superior biocompatibility and is suitable for use as an endodontic filling material.

## Figures and Tables

**Table 1:** ΔE_00_ results for the accelerated discoloration experiments.

	UV Exposure	0 s	2 s	5 s	15 s	1 min	3 min
Material							
Portland cement		0.0	1.5 ± 0.2	1.1 ± 0.5	1.3 ± 0.5	0.8 ± 0.5	2.1 ± 0.1
Bi_2_O_3_		0.0	3.3 ± 0.9	1.6 ± 0.3	10.8 ± 0.6	26.6 ± 1.1	35.3 ± 0.8
DI		0.0	1.0 ± 0.2	0.9 ± 0.1	1.1 ± 0.1	1.5 ± 0.1	1.5 ± 0.4
10% CaCl_2_		0.0	1.2 ± 0.2	0.9 ± 0.2	1.3 ± 0.2	1.5 ± 0.2	1.6 ± 0.2
20% CaCl_2_		0.0	1.3 ± 0.2	1.1 ± 0.2	1.4 ± 0.2	1.4 ± 0.2	1.3 ± 0.1

## Data Availability

Data are contained within the article.
